# Insanity

**Published:** 1884-02

**Authors:** 


### INSANITY.

The recent acquittal, on the ground of temporary insanity, of a young man charged with murder, and his subsequent examination and discharge by a commission of experts, recalls our attention to the subject of insanity, and leads us to express views long held, and which we believe can be maintained. The proposition which we make is as follows: Insanity, like most if not all other diseases not of traumatic origin, has no abrupt boundary lines. It merges more or less gradually into the ideally perfect mind on the one hand and the senseless idiot on the other. The ideally perfect mind, it has been truly said, does not exist; therefore, it follows that when, for practical purposes, the line must be drawn between what we will call practical sanity and incipient and slightly developed insanity, it is drawn arbitrarily, and the point upon which it is drawn depends equally upon the mind of the judge, his personal equation, and upon the mind of the judged.

This explains why we find that experts, equally qualified and honest, disagree—disagree so frequently that it is fast becoming proverbial. The sane of the one are the insane of the other. The melancholy or eccentric of the first party are the well-developed insane of the second. Thus, on this border-land, we have continual disagreement and contradiction. And from the nature of the case this, in a measure, must continue, if not for all time, at least until the pathological conditions of the brain and those indirectly effecting it are far more thoroughly mastered. What we desire to point out, however, is that this unavoidable fact has been ignored, in great measure, and that narrow and dogmatic opinions have taken the place of those wisely qualified. A correct appreciation of them, and observance of this fact, must inevitably result in good, first, because it is in accordance with pathology, and secondly, because with this correct appreciation must necessarily be associated correct expression. To decide dogmatically whether, in a certain doubtful case, the abnormality has proceeded to the point where it must be called insanity is as unscientific and unwise as to insist that at some well-defined and absolutely determined point hyperæmia is suddenly changed to inflammation. It is by insensible gradations that nature, no less in pathology than elsewhere, glides from one condition to another. Thus we see perfect vision changed to astigmatism, comparatively perfect digestion to imperfect digestion or dyspepsia; in like manner slowly develops the want of correspondence between assimilation and disassimilation—locally in pathological new formations, generally in atrophy or hypertrophy; and thus it is throughout the domains of physiology and pathology; they touch at all points, and merge insensibly into each other. Insanity is no exception. There is no line or point of demarcation, and to determine the exact point where sanity ends and insanity begins is impossible, for the very good reason that what does not exist cannot be discovered. No more ridiculous would it be to appoint a commission of experts to determine the exact points in the rainbow or solar spectrum where the red ends and the orange begins, where the indigo stops and the violet bounds it. The laws of refraction prevent the one; those of pathology, the other.

Instead of the expression, "this man is sane," or, "this man is insane," the truth of pathology and the demands of society can only be correctly satisfied by substituting an opinion somewhat like the following: "The person whom we have examined does, or does not, show sufficient mental aberration that, in our opinion or judgment, society and he require and are entitled to mutual protection."

The separate question of responsibility, morbid impulse, etc., we reserve for a future number.

